# Exploring the Feasibility of Suicide Prevention Intervention for Individuals With First Episode Psychosis Experiencing Suicidal Ideation: A Multi-Center, Mixed Method Study From Pakistan

**DOI:** 10.1192/bjo.2024.120

**Published:** 2024-08-01

**Authors:** Zaib un Nisa, Tayyeba Kiran, Jordan Bamford, Imran Bashir Chaudhry, Nusrat Husain

**Affiliations:** 1 Pakistan Institute of Living and Learning, Karachi, Pakistan; 2 Division of Psychology and Mental Health, Manchester, UK, Manchester, United Kingdom; 3 Zia ud Din university Hospital, Karachi, Pakistan

## Abstract

**Aims:**

First Episode Psychosis (FEP) emerges at a young age, significantly shaping the trajectory of the disorder. Literature indicates a 60% increased risk of suicide within the initial year of diagnosis in FEP, early intervention in psychosis reduces the risk of suicide. Therefore, this study aims to co-adapt an existing culturally appropriate suicide prevention intervention (CMAP) and integrate this with a culturally adapted Cognitive Behavioral Therapy for Psychosis (CaCBTp) for individuals with FEP experiencing suicidal ideation and to test its feasibility and acceptability in Pakistan.

**Methods:**

This is a mixed-method study that involves two stages. Stage 1 was co-adaptation of the CMAP intervention for people with FEP patients. This involved one-to-one, in-depth interviews with individuals with FEP (n = 5), carers (n = 5) and a focus group discussion with 10 healthcare professionals. The second stage involves feasibility testing of the intervention. Participants are being recruited (n = 90) from outpatient psychiatric units across the cities of Karachi, Lahore, Rawalpindi, Multan, and Hyderabad in Pakistan. Eligible, consented participants are being randomized into either of two trial arms; intervention arm or treatment as usual arm (TAU). All participants are being assessed at baseline and at 3-month post-randomization on assessing participants on severity of suicidal ideation, severity of symptoms, functionality and quality of life using different scales. The intervention is comprised of 12 one-to-one sessions delivered over 3 months by trained therapists. Participants (n = 15) from the intervention arm will be interviewed at the end of intervention to explore the acceptance.

**Results:**

Qualitative analysis of stage 1, utilizing thematic framework analysis, highlights barriers to help-seeking such as lack of awareness, inadequate social support, and mental health stigma. To adapt CMAP intervention, participants suggested changes in the use of Urdu words to make content simple for patients to understand, increase number of family sessions, include information about possible risk and protective factors of self-harm in this population and emphasize the addition of resilience-building messages in the manual. Stage 2 is currently ongoing, and we have successfully recruited healthcare facilities across all sites and randomized 12 participants into the trial.

**Conclusion:**

This study will add valuable insights for refinement of existing interventions to address the unique needs of individuals with FEP in Pakistan. Intervention with suicide preventive strategies may help in reducing the risk of suicide. The culturally grounded approach ensures relevance, contributing to the global discourse on evidence-based mental health interventions.

